# Field Application of the Micro Biological Survey Method for a Simple and Effective Assessment of the Microbiological Quality of Water Sources in Developing Countries

**DOI:** 10.3390/ijerph120910314

**Published:** 2015-08-25

**Authors:** Alyexandra Arienzo, Martin Sanou Sobze, Raoul Emeric Guetiya Wadoum, Francesca Losito, Vittorio Colizzi, Giovanni Antonini

**Affiliations:** 1 Department of Science, Roma Tre University, viale G. Marconi 446, 00146 Rome, Italy; E-Mails: alyexandraarienzo@gmail.com (A.A.); francescalos@hotmail.com (F.L.); 2 Department of Biomedical Sciences, University of Dschang, West Province, Cameroon; E-Mail: martinsobze@hotmail.com; 3 Department of Biochemistry, University of Dschang, West province, Cameroon; E-Mail: raoulemeric@yahoo.fr; 4 Department of Biology, University of Rome “Tor Vergata”, 00173 Rome, Italy; E-Mail: colizzi@uniroma2.it; 5 National Institute of Biostructures and Biosystems (INBB), 00136 Rome, Italy

**Keywords:** microbiological quality of water, water-borne diseases, MBS method

## Abstract

According to the World Health Organization (WHO) guidelines, “safe drinking-water must not represent any significant risk to health over a lifetime of consumption, including different sensitivities that may occur between life stages”. Traditional methods of water analysis are usually complex, time consuming and require an appropriately equipped laboratory, specialized personnel and expensive instrumentation. The aim of this work was to apply an alternative method, the Micro Biological Survey (MBS), to analyse for contaminants in drinking water. Preliminary experiments were carried out to demonstrate the linearity and accuracy of the MBS method and to verify the possibility of using the evaluation of total coliforms in 1 mL of water as a sufficient parameter to roughly though accurately determine water microbiological quality. The MBS method was then tested “on field” to assess the microbiological quality of water sources in the city of Douala (Cameroon, Central Africa). Analyses were performed on both dug and drilled wells in different periods of the year. Results confirm that the MBS method appears to be a valid and accurate method to evaluate the microbiological quality of many water sources and it can be of valuable aid in developing countries.

## 1. Introduction

Water is a complex and fragile element for both ecosystem and resources. Availability and access to safe drinking water is essential for human health and constitutes a basic right for all. According to the World Health Organization (WHO) guidelines “safe drinking-water must not represent any significant risk to health over a lifetime of consumption, including different sensitivities that may occur between life stages” [1]. Unfortunately this minimum quality standard is far from being reached, particularly in developing countries where water-borne diseases represent one of the first causes of death, especially of infants and young children. WHO estimates that 80% of diseases affecting the population of the planet are directly or indirectly related to water [2].

A good environmental monitoring system, operating within a robust legislative framework, is an essential prerequisite to identify sources of contaminants and build strategies to prevent these contaminants from entering into water sources that may be used for human consumption. Microbiological analysis carried out with traditional methods based on bacterial growth, and innovative methods based on genetic and/or enzymatic testing, are expensive or time-consuming and, more importantly, require skilled technicians and microbiological laboratories that are fully equipped with expensive instrumentation. Since sample integrity may be lost before it reaches a laboratory, monitoring needs to occur *in situ*, so that high quality data with excellent resolution can be collected for process studies and mapping. 

Alternative methods using portable, disposable and ready to use devices have been developed in order to meet the need to provide reliable results, thus diminishing the time of analysis, facilitating procedures and interpretation of data, increasing the detection sensitivity, limiting costs and allowing analysis in the absence of a laboratory. Their use is crucial in many situations and helps to overcome a great number of economic and logistic restrictions.

The aim of this work was the application of the Microbiological Survey (MBS) method for a simple, economic and effective microbiological analysis of drinking water. The MBS method is an alternative, fast, colorimetric system for microbiological analysis developed and patented by Roma Tre University, Italy. The MBS analytical kit is made of disposable, ready to use reaction vials for microbiological analysis that measure the catalytic activity of redox enzymes of the main metabolic pathways of bacteria, allowing an unequivocal correlation between the observed enzymatic activity and the number of total viable cells in the samples. The analysis is based on the colour change of the vial content that is induced by the presence of bacteria. The colour change can be simply monitored by visual inspections and the time required for colour change is inversely related with the number of viable bacteria in the analysed samples by means of specific correlation tables. Like an enzymatic reaction, the greater the number of bacteria the faster will be the colour change. The validation of the MBS method for the analysis of artificially contaminated water samples according to IS0 13843 has already been carried out and published [3,4,5].

Currently available detection methods do not allow for the routine analysis of all microorganisms that could be present in inadequately treated drinking water. The evaluation of total heterotrophic bacteria and total coliforms are two among the parameters used for monitoring the overall quality of drinking water. Heterotrophs are broadly defined as microorganisms that require organic carbon for growth and their measurement is used to indicate the effectiveness of water treatment processes and to investigate aesthetic quality [6]. In rural areas this parameter is also used as indicator for the potential presence of opportunistic pathogens in water [7]. Total coliforms are a group of bacteria that are widespread in nature. All members of the total coliform group can occur in human feces, but some can also be present in animal manure, soil, and submerged wood and in other areas outside the human body. For drinking water, total coliforms are used to determine the adequacy of water treatment and the integrity of the distribution system. The absence of total coliforms minimizes the likelihood that faecal pathogens are present and are therefore used as indicator of faecal contamination and to determine the vulnerability of a system to contamination by any outside source [8].

The need to assess the microbiological quality of water sources is particularly important in Douala, Cameroon, where water-related diseases account for about two-thirds of all recorded diseases and are responsible for about 50% of the reported cases of death. About 3.8 million Cameroonians lack access to adequate sanitation [9]. Sanitation in the African cities (60%–95%) is generally dominated by the autonomous systems (SAA) (WC + septic tanks, latrines, *etc*.). The effluents of the SAA are rich in coliforms and faecal helminths, viruses, protozoa and in various chemical and physical pollutants [10]. The intrusion of these faecal effluents in the aquifers or distribution systems can generate various diarrheal diseases in the human population and contribute to a great part in the deterioration of public health [11]. In Douala nearly 60% of resident populations collects groundwater through wells and springs [12]. Dug wells are a traditional method of obtaining water, which have been used for thousands of years. In rural areas these are open and unprotected and therefore can be easily contaminated from spilt water, animal excreta and other environmental pollutants. Dug wells in these conditions represent a major risk to public health and therefore contribute to the spread of waterborne diseases. Drilled wells can be excavated by simple hand drilling methods (augering, sludging, jetting, driving, hand percussion) or machine drilling (rotary, percussion, down the hole hammer). Drilled wells with electric pumps are used throughout the world, typically in rural or sparsely populated areas, though many urban areas are also supplied partly by municipal wells. Drilled wells can get water from a much deeper level than dug wells and can often be up to several hundred meters deep, typically ranging from 3 to 18 m to more than 900 m. These characteristics make drilled wells generally considered safer than the dug ones. This paper demonstrates the possibility of successfully using the MBS method for a simplified and effective microbiological analysis of drinking water.

## 2. Experimental Design

### 2.1. Preliminary Study

#### 2.1.1. Samples Collection

Fifty naturally contaminated water samples were collected from domestic (ten samples) and industrial (ten samples) distribution systems and from water wells (30 samples) of an agricultural region located in the south of Rome (Province of Latina), Italy. Water samples were collected in appropriate sterile bottles, immediately stored in a refrigerator box and transported to the laboratory for analysis. Thiosulfate was added, before analyses were performed, to the water samples treated with chlorine.

#### 2.1.2. MBS Method Procedure for the Analysis of 100mL of Water 

Analysis of 100 mL of water with the MBS method was carried out using Total Viable Count (TVC) and COLIFORM vials for the detection and quantification of mesophilic aerobic bacteria or TVC and total coliforms, respectively. The MBS analysis of 100 mL of water samples was performed using a filtering device made of a polycarbonate body provided with a polycarbonate filter of 0.45 µm. The filtering device was assembled and sterilized in autoclave at 121 °C for 15 minutes and then used as a normal syringe filter. One hundred mL of water samples were filtered through the filtering device and the filter was extracted from the polycarbonate body using a sterile nipper and then inserted into the MBS vials previously filled with 10 mL of sterile distilled water. After the insertion of the filter, TVC and COLIFORM vials were incubated at 37 °C for 30 hours in an optical reader that automatically detects the change of colour of the vials.

#### 2.1.3. Reference Method of Membrane Filtration 

The analysis of water samples with the reference method of membrane filtration was performed using filters with pores of 0.45 µm: 100 mL of water samples were filtered using the apposite membrane filtration system and filters were incubated on Plate Count agar at 37 °C for 48 hours for Total Viable Count and on MacConkey agar at 37 °C for 24 hours for total coliforms detection. The results were obtained by standard plate counting methods with deduction ranging from 30–300 colonies per plate.

#### 2.1.4. Linearity and Accuracy of the MBS Method 

Water samples coming from domestic and industrial distribution systems and from private wells were analysed both with the MBS method and with the reference methods. The results were then compared in order to demonstrate linearity and accuracy. The parameters examined were Total Viable Count at 37 °C and total coliforms in 100 mL of sample water.

#### 2.1.5. Correlation between the Analysis of 1 mL and 100 mL Water Samples 

One hundred mL and 1 mL water samples were analysed with the MBS method and the reference methods, respectively. For the MBS method 100 mL were analysed as previously described using COLIFORM vials; 1 mL was analysed simply inoculating COLIFORM vials with 1 mL of water samples. COLIFORM vials were incubated at 37 °C for 30 hours in an optical reader that automatically detects the change of colour of the vials. For the analysis of 100 mL the membrane filtration technique was used as the reference method, *i.e.*, water samples were filtered and the filters were incubated on MacConkey agar at 37 °C for 24 hours. For the analysis of 1mLthe Plate count technique was used: 1 mLof the samples was plated on MacConkey agar at 37 °C for 24 hours. The results were obtained by standard plate counting methods with deduction ranging from 30–300 colonies per plate and were compared in order to verify a possible correlation.

### 2.2. In situ Application of the MBS Method 

#### 2.2.1. The Study Area

Douala, the economic capital of Cameroon, is located between 4°04' latitude north and 9°45' longitude east, and it is situated near the Atlantic coast 1 m above sea level within the Congo-Guinean phytogeographic zone [13]. It is characterized by a typical warm and humid equatorial climate, with an average annual temperature of 27.0 °C (80.6 °F), an average humidity of 85% and two rainy seasons extending from March to June and from September to November. The soil is made up of coastal sands, black mud of mangrove swamps and fluvial deposits. 

#### 2.2.2. Data Collection

Data were collected in three different phases in order to study the effects of inter-annual meteorological variation of rainfall and temperature on groundwater characteristics. The first phase took place in November 2012, within the rainy season, characterized by heavy and frequent precipitations and by a moderate temperature of 30 °C–38 °C. The second phase took place in January 2013, when the rainfalls were absent and the temperature was very high, with an average of 40 °C–45 °C. The third phase took place in March 2013, at the beginning of the rainy season and at high temperatures of 40 °C–45 °C.

Sixty one water points were selected within 20 quarters of the Douala V City council. The water points were divided into dug wells and drilled wells and all of them were used as drinking water sources by the resident population. All the dug wells were between 1.5 and 6 m deep while drilled wells were all deeper than 20 meters.

After taking the GPS (LG Marquee^™^, Seoul, South Korea) coordinates of the water points, water samples were collected in 50 mL sterile plastic containers (Falcon tubes, BD, San Jose, CA, USA), treated with thiosulfate to inhibit the antimicrobial activity of the chlorine eventually used to treat water, immediately stored in a refrigerator box and transported to the field lab, set up in the Douala V City council, for analysis. The period between the sample collection and the analysis in all cases was less than 6 hours. All wells were analysed in each phase and for each sample analyses were performed in triplicate.

#### 2.2.3. MBS Method Procedure for Quantitative Evaluation of Total Coliforms 

Analysis with the MBS method was carried out using COLIFORM vials for the detection and quantification of total coliforms. To carry out analyses, the vials were filled with 10 mL of sterile distilled water and 1 mL of the samples was added to each vial. After inoculation the vials were incubated at 37 °C for 24 hours in a thermostat. The change of colour was checked at two different time intervals corresponding to three levels of contamination: 14 and 24 hours. A colour change to yellow after 14 hours indicates a high contamination (total coliforms concentration higher than 100 CFU/mL), a colour change to yellow within 24 hours indicates contamination (total coliforms concentration lower than 100 CFU/mL) and no colour change after 24 hours indicates no contamination (absence of total coliforms). Independent analyses using traditional methods were carried out by the “Louis Pasteur Labo” (“Laboratoire Multidisciplinaire d’Analyses de Biologie Médicale”).

#### 2.2.4. Georeference

Georeference was carried out using the Oziexplorer software (ver 3.95.5r) by D&L Software Pty Ltd.(Brisbane Australia), licensed to G.A. Douala map was downloaded from Google Maps. Geographical coordinates refer to WGS 84 Datum.

## 3. Results

The first part of the study was developed to demonstrate that in naturally contaminated water samples, the analysis with the MBS method of total coliforms present in 1 mL of water is a reliable measure for general assessment of microbiological contamination of water.

### 3.1. Linearity and Accuracy of the MBS Method

The MBS analysis was performed on 100 mL of 50 water samples as previously described using two different MBS vials, TVC MBS vials for Total Viable Count and COLIFORM MBS vials for the detection of total coliforms. The same samples were also analysed with the reference method of membrane filtration.

**Table 1:** Comparison analysis of the results obtained both for Total Viable Count and for total coliforms in 100 mL of different water samples between the MBS method and the reference method.

MBS Method	Reference Method		
	Present	Absent	Total
Positive	61 (PA)	0 (PD)	61
Negative	0 (ND)	39(NA)	39
Total	61 (N_+_)	39 (N_-_)	100(N)

PA: positive agreement, NA: negative agreement, ND: negative deviation (false negatives), PD: positive deviation (false positives), N: total number of samples (NA+PA+PD+ND), N_+_: total number of positive results obtained with reference method, N_-_: total number of positive results obtained with reference method.

A comparative presence/absence analysis was carried out: of the 100 analyses performed, 61 were positive with both methods without the presence of false negatives (sensitivity 100%) and 39 were negative with the total absence of false positives (specificity 100%) (Table 1). 

**Figure 1:**
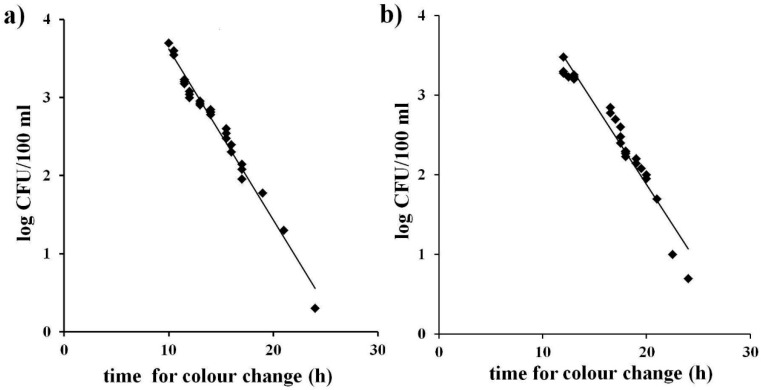
Correlation between the bacterial concentrations obtained by reference methods (log of CFU/mL) and the time occurring for colour change in the MBS vials. (**a**): Total Viable Count; (**b**): total coliforms. The straight line represents the linear regression analysis. Each point is the mean of three different analyses.

Linearity is the ability of the method when used with a given matrix to give results that are in proportion to the amount of analyte present in the sample, that is, an increase in analyte corresponds to a linear or proportional increase in results as indicated by ISO 16140 [14]. This was achieved graphically as illustrated in Figure 1 a, b by plotting bacterial concentrations (expressed as the log of CFU/mL) obtained with the reference method with the time occurring for colour change of the identical water samples analysed with the MBS method both for TVC and for total coliforms. A linear inverse relationship between the MBS method and the bacterial concentration can be observed in both cases. The equations of the curves and their correlation factors (R^2^) were calculated (0.98 for Total Viable Count and 0.94 for total coliforms). The equations of the curves were used to determine, from the time occurring for colour change, the bacterial concentrations (expressed as the log of CFU/mL) so as to demonstrate the accuracy of the MBS method.

**Figure 2:**
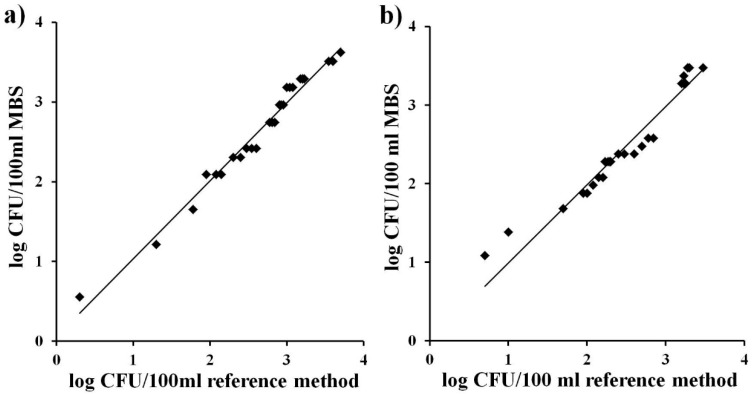
Correlation between bacterial concentrations (log of CFU/mL) obtained with the MBS method and with the reference method on identical samples of naturally contaminated water. (**a**) Total Viable Count; (**b**) total coliforms. The straight line represents the linear regression analysis. Each point is the mean of three different analyses.

Accuracy is the degree of correspondence between the response obtained by the reference method and the response obtained by the alternative method on identical samples (ISO 16140). A perfect correlation between the bacteria number (expressed as log CFU/mL) obtained with the traditional method of membrane filtration and the alternative MBS method for the different water samples was observed both for TVC and for total coliforms (Figure 2a and Figure 2b). The straight lines obtained were close to the ideal y = x (slope = 1.00), with values of correlation factor (R^2^) which further confirm the high equivalence between the reference method and the alternative MBS method (Slope = 0.98, R^2^= 0.98for Total Viable Count; Slope = 0.99, R^2^ = 0.94 for total coliforms).

### 3.2. Correlation between Total Viable Count at 37 °C and Total Coliforms on 100 mL of Water Samples

To further simplify the analysis, a comparative study between the two parameters, Total Viable Count and total coliforms, in different water samples was carried out. Coliforms are facultative anaerobic, Gram-negative, non-spore forming rods that ferment lactose. They are commonly used as bacterial indicators of sanitary quality of water and considered as a general indicator of contamination [15].

**Figure 3:**
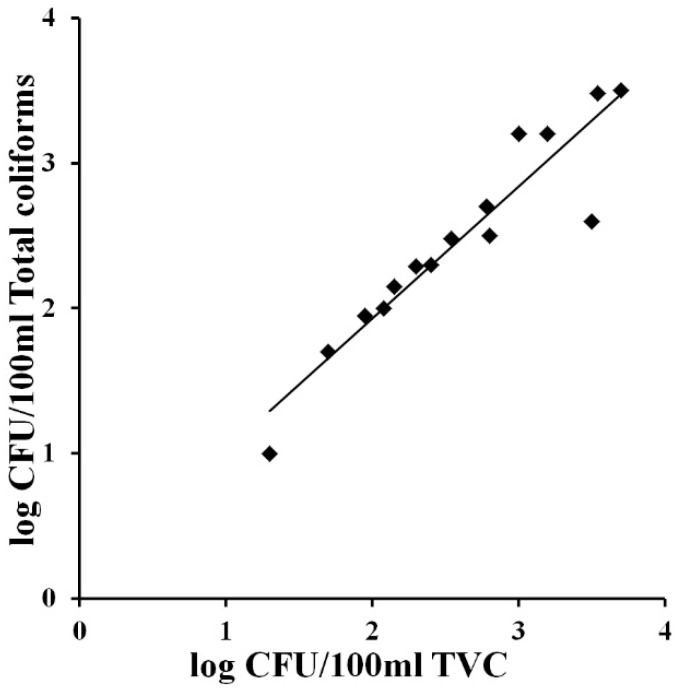
Correlation between total Viable Count and total coliforms concentrations (log of CFU/100 mL) in identical water samples from domestic and industrial distribution systems. Bacterial concentrations were obtained with the MBS method. The straight line represents the linear regression analysis. Each point is the mean of three different analyses.

The correlation between the two parameters was achieved graphically by plotting the concentration of Total Viable Cells (expressed as the log of CFU/100 mL) against the concentration of total coliforms obtained with the MBS method. Figure 3 shows that in the presence of total coliforms a strict correlation between the two parameters can be observed (Slope = 0.91, R^2^ = 0.87). This result confirms the possibility of using only the parameter total coliforms to have a good evaluation of the microbiological quality of water. Total coliform count is in fact the main bacteriological parameter that has been used to determine the general quality of drinking water worldwide [16]. This test could therefore be used as the starting point to determine the biological quality of drinking water. In particular the total coliforms test is considered as an indicator, since the presence of bacteria in this group indicates the possibility, but not the certainty, that pathogenic organisms may also be present in the water.

### 3.3. Correlation between the Analysis of100mLand 1mLof Water Samples 

According to international standards some parameters must be evaluated in 100 mL of water samples. The possibility to apply the MBS method for the analysis of 100 mL of water has been successfully demonstrated. However, the realization of this type of analysis requires minimal equipment and is more laborious and time consuming. Because the next step of the study considered the use of the MBS method on field and in particularly difficult conditions, a preliminary study to verify the margin of error between the analysis of 1mLand the analysis of 100 mL was carried out. This can be also very important for turbid water and water with suspended particles that is difficult to filter. After the demonstration of the good correlation between the results obtained analysing the same samples with both the reference method and the MBS method (data previously shown), the MBS results obtained from the analysis of 1 mL of the sample were normalized for 100 mL and then compared to the results obtained analysing 100 mL.

**Figure 4:**
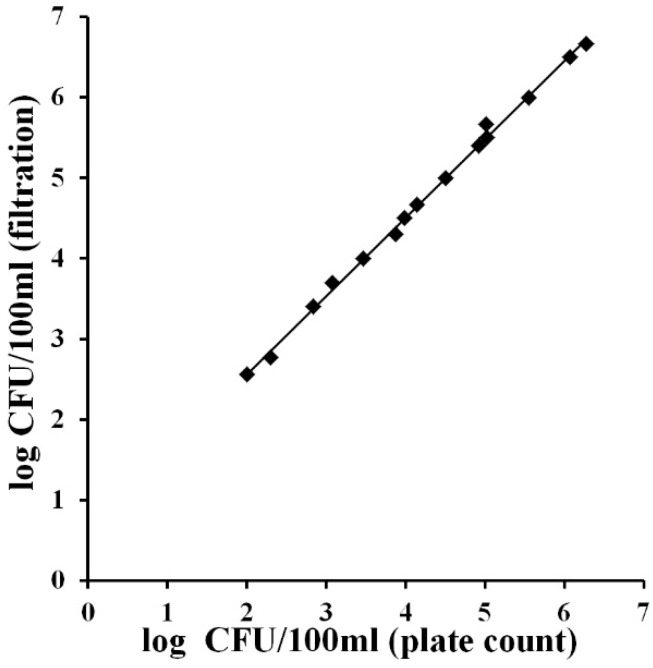
Correlation between total coliform concentrations (log of CFU/100 mL) obtained using the MBS method for the analysis of 1 mL and 100 mL of identical water samples. The straight line represents the linear regression analysis. Each point is the mean of three different analyses.

Figure 4 shows that in the presence of a concentration of coliforms greater than 10^2^ the analysis of 1 mL underestimates the bacterial concentration of 0.5 log units (Slope = 0.97, R^2^ = 0.99). This result can be acceptable considering that the Most Probable Number technique, a standard method used for water analysis, has a given uncertainty of about 1 log unit.

### 3.4. In situ Application of the MBS Method 

The MBS method was then applied to evaluate the microbiological quality of water in the city of Douala. According to the results obtained in the preliminary studies the concentration of total coliforms was enumerated in 1mL of water samples. Absence of coliforms, required by law, can be simply assessed monitoring the colour change of the vials after 24 hours. However, in order to distinguish three levels of contamination the change of colour was monitored visually at two different time intervals (Table 2).

**Table 2:** Water contamination in the city of Douala determined by the time of colour change of the COLIFORM MBS vials and by the total coliform concentrations expressed in CFU/mL.

Colour after 14 hours	Colour after 24 hours	Bacterial Concentration (CFU/mL)
Yellow	Yellow	>10^3^ = high contamination
Yellow	Red	1< x< 10^3^= contamination
Red	Red	No contamination

**Figure 5:**
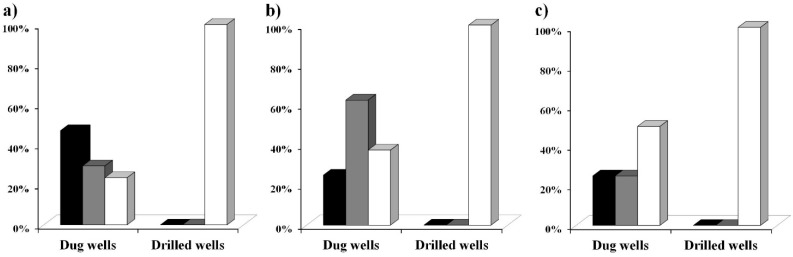
Coliform contamination of water samples from drilled wells and from dug wells. The type of water point and the coliform contamination level of the water samples tested with the MBS method are shown together with the examination phase. Black bars mean high contamination by coliform (>100 CFU/mL). Grey bars mean low coliform contamination (between 1 and 100 CFU/mL). White bars mean no coliform presence. Data were collected in three periods: (**a**) November 2012, (**b**) January 2013, (**c**) March 2013.

Figure 5 shows the level of coliform contamination found in the water samples analysed with the MBS vials for total coliforms in the three different phases. From the obtained data we have assessed that in the first phase 70% of the dug wells analysed were contaminated while 100% of the analysed drilled wells were not contaminated. 

One water sample was collected from a natural source and it was found to be highly contaminated. This first phase took place in November 2012, within the rainy season, characterized by heavy and frequent precipitations and by a moderate temperature of 30 °C–38 °C. Very similar results were obtained during the second phase that took place in January 2013, when the rainfalls were absent and the temperature was very high, with an average of 40 °C–45 °C: 62.5% of the dug wells analysed in this second phase were contaminated while 100% of the analysed drilled wells were not contaminated. During the third phase that took place in March 2013, when the rainfalls begin and the temperature was still very high, with an average of 40 °C–45 °C, it resulted that 50% of the analysed dug wells were contaminated while 100% of the analysed drilled wells were not contaminated. Independent analysis performed by the “Louis Pasteur Labo” (“Laboratoire Multidisciplinaire d’Analyses de Biologie Médicale”) confirmed the absence of coliforms contamination in some of the drilled wells analysed with the MBS method (data not shown). 

In addition, out of 28 samples that resulted positive for total coliforms contamination in the first phase, 83% of the samples also tested positive for *Escherichia coli* indicating a recent faecal contamination and the high level of danger of the water samples.

**Figure 6:**
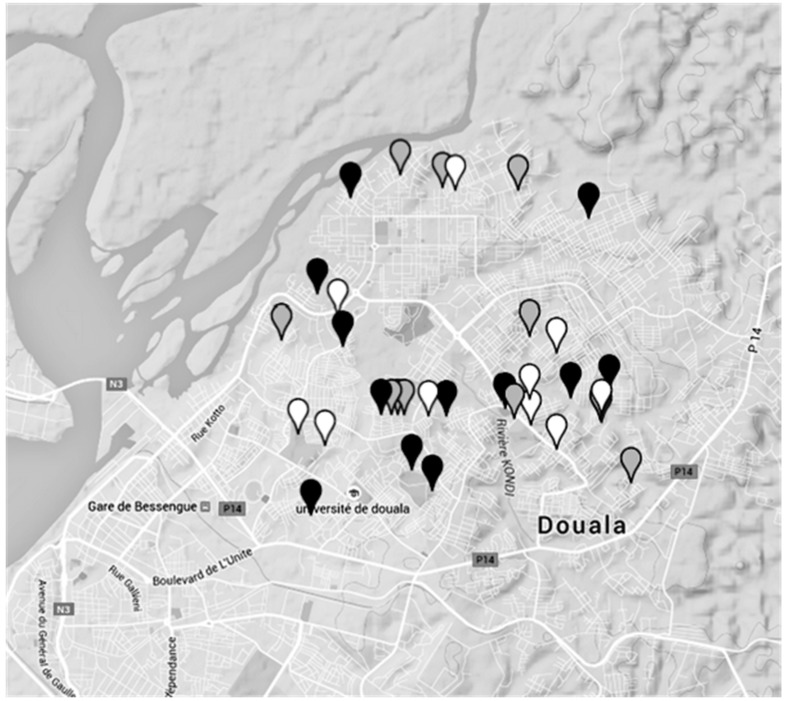
Contamination levels of dug wells in Douala (Cameroon).The colour of the water points refers to the highest level of coliform contamination of the water samples found with the MBS method. Black spots mean high contamination by coliform (>100 CFU/mL). Grey spots mean low coliform contamination (between 1 and 100 CFU/mL). White spots mean no coliform presence.

Figure 6 shows in particular the different levels of contamination of dug wells. For each spot the highest level of contamination found among the three phases was chosen.

## 4. Discussion

Groundwater represents one of the principal sources of fresh water and hence it plays an enormous importance in domestic and industrial activities. In view of the groundwater being used for drinking purposes, its quality remains one of the major issues of concern. Groundwater is an aquatic biotope that contains a varied microflora. The use of water from wells, without any previous treatment, can involve serious health problems due to the potential presence of pollutants and pathogenic bacteria [17]: unhygienic peridomestic sanitation and unsafe environments lead to the incidence of waterborne illness and place children at risk of death [18]. Water from such sources, in fact, seldom complies with WHO’s permissible standard limits for drinking water [19].

The use of a fast, easy and cheap method for microbiological analysis that can be used by a non-skilled technician and without a fully equipped microbiological laboratory is of fundamental importance for the management of safe drinking water sources, especially in developing countries. In this study it was demonstrated that the evaluation of total coliforms present in 1 mL of water carried out using the colorimetric MBS method, is sufficient for a rough, though important, evaluation of the microbiological quality of water samples. The presence of total coliforms in drinking water is enough to assume that a potential health hazard exists because of the possible presence of pathogens since total coliforms are the general indicator of faecal contamination [15]. 

A significant difference in coliform concentration levels between the two types of wells was observed. In Douala, 69.6% of the examined dug wells were contaminated by total coliforms with an average contamination that exceeded the microbiological standards for drinking water recommended by the World Health Organization (WHO), which are 0 CFU/100 mL for total coliforms. On the other hand, 100% of drilled wells resulted non contaminated, underlining the safeness of these water sources and the importance of their use in both rural and city areas.

It can be seen from Figure 6 that no connection between the level of coliform bacteria contamination and the position of the water points was observed. However, it is important to underline that during the rainy season the number of highly contaminated water samples increases from 25% (observed in dry periods) up to 41%. This can be explained by the fact that this water resource is vulnerable not only to anthropogenic pollution but also influenced by the effects of rainfalls which vary not only throughout the year but also fluctuate from year to year. The heavy and frequent rainfalls, and the higher level of the phreatic layer of groundwater, observed in November could be responsible for the higher levels of contamination due to the greater possibility of infiltration, draining or streaming of water from septic pits [20].

Unfortunately, dug wells remain one of the most sought after and easily accessible water sources. Based on these findings, it is prudent to frequently monitor the microbiological safeness of these water sources and identify the links between water quality, environmental quality, sanitation and public health. These relations are under investigation by a specific survey performed by the students of the Public Health Master School in the University of Dschang that will provide further information.

## 5. Conclusions

Environmental monitoring results are essential to identify sources of contaminants and build strategies to avoid these from entering water resources that may be used for human consumption. The training of suitable personnel, together with the development of systems for data validation and quality assurance, are equally important. This requires people to operate and maintain the instrumentation and others to interpret the data, formulate policy and implement strategy. 

The MBS method appears to be a valid and accurate method to frequently evaluate the microbiological quality of many water sources and its simple procedure and interpretation of results can be suitable for its use in rural areas by local trained personnel operating without a microbiological laboratory. The field application of the MBS method for the analysis of water quality in Douala supported the hypothesis that drinking water coming from natural sources or dug wells is frequently contaminated, while water obtained from drilled wells is never contaminated, suggesting that all the dug wells should be frequently monitored or discontinued in favour of safer drilled wells. 
